# Difference of 11 years between two periods of VV-ECMO does not impact mortality in large centres: we are not sure

**DOI:** 10.1186/s13054-022-03952-y

**Published:** 2022-05-23

**Authors:** Patrick M. Honore, Sebastien Redant, Pharan Djimafo, Thierry Preseau, Bogdan Vasile Cismas, Keitiane Kaefer, Leonel Barreto Gutierrez, Sami Anane, Andrea Gallerani, Rachid Attou

**Affiliations:** 1 ICU Department, Centre Hospitalier Universitaire Brugmann-Brugmann University Hospital, Place Van Gehuchtenplein, 4, 1020 Brussels, Belgium; 2 ED Department, Centre Hospitalier Universitaire Brugmann, Brussels, Belgium

**Keywords:** VV-ECMO, Influenza H1N1 in 2009, COVID-19 in 2021, Difference of 11 years, Impact upon mortality, COVID-19, H1N1, Influenza, ARDS, ECMO

Fanelli et al. conclude that in patients with acute respiratory distress syndrome (ARDS) who received extracorporeal membrane oxygenation (ECMO), the observed unadjusted 60-day mortality was higher in cases of COVID-19 than influenza H1N1 pneumonia [1]. This difference in mortality was not significant after multivariable adjustment; older age and longer hospital length of stay before ECMO emerged as important covariates that could explain the observed difference [1]. The authors state that the current study has several limitations including the possible effect of change of standard of care on outcomes compared over a period of more than 10 years [1]. However, they state that selected academic ECMO centers with high volume and experience in extracorporeal support should guarantee a high standard of care in treating both COVID and influenza H1N1 patients [1]. We would like to comment.The statement that the outcome of ECMO should be the same in 2009 versus 2021 in selected academic ECMO centers is contradicted by the literature [2]. Numerous studies have shown that an enormous evolution has occurred in techniques over a decade [2]. A relatively recent meta-analysis which compares mortality rates over a period of 15 years (from 2000 up to 2015) clearly showed a reduced mortality after adjustment for confounding variables [2]. The meta-regression model in this study showed that the year of study realization was an independent risk factor for mortality (*b* =  − 0.176; *p* = 0.003) [2, 3]. Undoubtedly findings would be similar for the period between 2009 and 2021, an 11 year period in which ECMO has evolved enormously [2, 3]. A meta-analysis by Vaquer et al. demonstrated that the impact of cannula size is an independent factor for mortality (*b* =  − 0.075; *Q* = 7.04; *n* = 4; *p* = 0.008) [2]. In the study by Fanelli, veno-venous (VV) ECMO blood flow and sweep gas flow were similar in the two groups, except for higher blood flows at day 1 and 14 in the COVID-19 group [1] possibly confirming the role of increased cannula size with increased flow over the years [2]. Another recent study confirmed that a larger drainage cannula diameter was also associated with a reduced risk of death on ECMO [HR 0.88 (0.80–0.96), *p* = 0.005] [4]. Progressive technological evolution of VV-ECMO equipment, with improved biocompatibility and reduced complication rates, could have had a potential positive impact on patient evolution [3]. Finally, centre experience has also been associated with improved outcomes in previous reports [3].

## Data Availability

Not applicable.

## Abbreviations

ARDS: Acute respiratory distress syndrome
ECMO: Extracorporeal membrane oxygenation
VV-ECMO: Veno-venous ECMO
