# Supplementary material for: Difference of 11 years between two periods of VV-ECMO does not impact mortality in large centres: we are not sure
Source: Crit Care. 2022 May 23;26:149. doi: 10.1186/s13054-022-03952-y (PMC9125954; doi:10.1186/s13054-022-03952-y)
Supplement: Related texts

# Difference of 11 years between two periods of VV-ECMO does not impact mortality in large centres: we are not sure. Authors’ reply

**Authors:** Vito Fanelli, Marco Giani, Giacommo Grasselli, Luca Brazzi, Marco V. Ranieri

**Affiliations:** 3 grid.7605.4 0000 0001 2336 6580 Department of Surgical Sciences, University of Turin, Turin, Italy; 4 grid.7605.4 0000 0001 2336 6580 Department of Anesthesia, Critical Care and Emergency - Città della Salute e della Scienza Hospital, University of Turin, Corso Dogliotti 14, 10126 Turin, Italy; 5 grid.7563.7 0000 0001 2174 1754 Department of Emergency and Intensive Care, School of Medicine and Surgery, ASST Monza, University of Milano-Bicocca, Monza, Italy; 6 grid.414818.0 0000 0004 1757 8749 Department of Anesthesia, Critical Care and Emergency, Fondazione IRCCS Ca’ Granda Ospedale Maggiore Policlinico, Milan, Italy; 7 grid.6292.f 0000 0004 1757 1758 Alma Mater Studiorum – Università di Bologna, Dipartimento di Scienze Mediche e Chirurgiche (DIMEC), Anesthesia and Intensive Care Medicine, IRCCS Policlinico di Sant’Orsola, Bologna, Italy

We thank Dr. Honore and colleagues for their comments to our study on extracorporeal membrane oxygenation (ECMO) support for COVID-19 and H1N1 patients with ARDS [1]. They raise few points that we want to address. Over the last decade, the care of critical ARDS patients on ECMO support has indeed evolved. Dr Honore and colleagues argue that the outcome of ARDS patients treated with ECMO has improved overtime given the technological improvement of extracorporeal support. Recent evidences show that greater attention has been paid on limiting driving pressure to minimize the risk of ventilator induced lung injury [5]. Ultraprotective ventilation was already implemented in our H1N1 ECMO cohort, as shown by a median driving pressure of 10 (see electronic supplement), which was lower compared to COVID-19 patients. In addition, the use of prone positioning has become a standard of care after 2013 [6] but, it has been implemented in patients on ECMO support only in the last few years and was frequently used in COVID-19 patients. This might have influenced our findings, as two recent large meta-analysis on retrospective studies showed a possible association with reduced mortality [7, 8]. Moreover, in our H1N1 cohort hospital mortality was around 30%, similar to the rate reported in the EOLIA trial [9] and did not improve over time. Dr Honore and colleagues speculate that higher blood flow at day 1 and 14 in COVID-19 group might be related to the use of larger cannula over time, which was reported to be associated with higher mortality. In our study, the hazard ratio of death at 60 days was estimated accounting for blood flow and sweep gas at day 1 of extracorporeal support. The hazard ratio of death at 60 days decreased from 1.76, (95% CI 1.17–2.64) to 1.54 (95% CI 0.82–2.90) accounting for blood flow and sweep gas at day 1 of extracorporeal support; only adjusting for confounders, mainly age and hospital length of stay before ECMO support, the hazard ratio decreased noticeably to 1.39, 95% CI 0.78–2.47, suggesting that patients characteristics at baseline primarily accounted for the higher risk of death of COVID-19 patients. Furtheremore, Dr Honore and colleagues argue that center experience affects patients outcome. This is certainly reasonable, however in the current study the H1N1 and COVID-19 cohorts were enrolled at the same ECMO centers and the hazard ratio of death at 60 and 90 days were all estimated stratifying by center-level. We are aware that it is impossible to conclusively resolve in one study all the factors influencing the outcome of ECMO patients, especially over twelve years study period. Nonetheless, we believe that our study provides useful insights for clinicians who deal with the difficult task of defining the indication for ECMO and shows that outcome depend on patient selection rather than the different viral etiology.
